# Are national policies and programs for prevention and management of postpartum hemorrhage and preeclampsia adequate? A key informant survey in 37 countries

**DOI:** 10.9745/GHSP-D-14-00034

**Published:** 2014-07-03

**Authors:** Jeffrey Michael Smith, Sheena Currie, Tirza Cannon, Deborah Armbruster, Julia Perri

**Affiliations:** a Jhpiego, MCHIP, Washington, DC., USA; b University of Washington, Seattle, WA., USA; c United States Agency for International Development, Washington, DC., USA; d Jhpiego, Baltimore, MD., USA

## Abstract

**Introduction:**

Although maternal mortality has declined substantially in recent years, efforts to address postpartum hemorrhage (PPH) and preeclampsia/eclampsia (PE/E) must be systematically scaled up in order for further reduction to take place. In 2012, a key informant survey was conducted to identify both national and global gaps in PPH and PE/E program priorities and to highlight focus areas for future national and global programming.

**Methods:**

Between January and March 2012, national program teams in 37 countries completed a 44-item survey, consisting mostly of dichotomous yes/no responses and addressing 6 core programmatic areas: policy, training, medication distribution and logistics, national reporting of key indicators, programming, and challenges to and opportunities for scale up. An in-country focal person led the process to gather the necessary information from key local stakeholders. Some countries also provided national essential medicines lists and service delivery guidelines for comparison and further analysis.

**Results:**

Most surveyed countries have many elements in place to address PPH and PE/E, but notable gaps remain in both policy and practice. Oxytocin and magnesium sulfate were reported to be regularly available in facilities in 89% and 76% of countries, respectively. Only 27% of countries, however, noted regular availability of misoprostol in health facilities. Midwife scope of practice regarding PPH and PE/E is inconsistent with global norms in a number of countries: 22% of countries do not allow midwives to administer magnesium sulfate and 30% do not allow them to perform manual removal of the placenta.

**Conclusions:**

Most countries surveyed have many of the essential policies and program elements to prevent/manage PPH and PE/E, but absence of commodities (especially misoprostol), limitations in scope of practice for midwives, and gaps in inclusion of maternal health indicators in the national data systems have impeded efforts to scale up programs nationally.

## Abstract (precis)

Most surveyed countries have many supportive policies and program elements, but issues remain that impede maternal health efforts, including: inconsistent availability of essential commodities, particularly misoprostol; limitations on midwives' scope of practice; incomplete or out-of-date service delivery guidelines; and weak reporting systems.

## INTRODUCTION

In 2010, approximately 287,000 women worldwide died of pregnancy-related causes—a decline of 47% since 1990.^1^ Despite this considerable progress, maternal mortality remains unacceptably high in many countries, with sub-Saharan Africa and South Asia having the greatest burden of maternal death.

Efforts to reduce maternal mortality have included attention to the 2 leading causes: postpartum hemorrhage (PPH) and preeclampsia/eclampsia (PE/E). Global recommendations point national maternal health programs to a set of key components that should be addressed to successfully reduce maternal morbidity and mortality:

Postpartum hemorrhage and preeclampsia/eclampsia are the 2 leading causes of maternal death.

- The presence of oxytocin, misoprostol, and magnesium sulfate, in correct dosages, on the World Health Organization (WHO) and national essential medicines lists^2^
- The need to ensure that these commodities are available in sufficient quantities and stored correctly at health facilities^3^
- Policy and service delivery guidelines that support the provision of uterotonics (including use of active management of the third stage of labor [AMTSL]) for the prevention of PPH,^4^ the use of misoprostol at home birth when AMTSL is not possible,^4^ and the use of magnesium sulfate in the management of severe PE/E^5^ by the appropriate categories of personnel
- Legal authorization, or authorization through national guidelines, for midwives to administer oxytocin and magnesium sulfate and to perform manual removal of placenta, as well as their education in these practices^6^ or task shifting, as may be needed for advanced distribution of misoprostol
- Inclusion of up-to-date, evidence-based guidelines as the basis of in-service training and preservice education^7^^,^^8^
- Monitoring, evaluation, and reporting on the provision of uterotonics as a national indicator^4^

The extent to which these interventions are in place in a country indicates the likelihood that a country will be addressing their major causes of maternal mortality.

With this in mind, the Maternal and Child Health Integrated Program (MCHIP), with support from the United States Agency for International Development (USAID), undertook an augmented key informant survey in 2012 of national health programs supported by USAID in 43 countries, especially those facing the highest burden of maternal mortality. The goal of this multi-country survey was to provide a global snapshot of the extent to which these essential policies and programs were in place and to provide program managers and development partners with evidence on the key processes that facilitate scale up and expansion of maternal health interventions, especially evidence-based PPH and PE/E program interventions. This article summarizes the most relevant findings from the 2012 survey. The full report, including the questionnaires (in English, French, and Spanish), is available at: www.mchip.net/globalstatusreportdownloads.

## METHODS

Between January and March 2012, we conducted a key informant survey of national programs for the prevention and management of PPH and PE/E. The survey consisted of a 44-item questionnaire that addressed 6 core programmatic areas: policy, training, medication distribution and logistics, national reporting of key maternal health indicators, programming, and challenges to and opportunities for scale up. (See the supplementary material for the survey instrument.) The majority of questions required a dichotomous yes/no response, while some required responses on a graded scale. Qualitative open-ended questions were asked in 2 areas: programming and challenges to and opportunities for scale up. All questions included the option to provide additional explanation, and some specifically requested more information, depending on the response. Professional translators translated the survey instruments from English into French and Spanish, and assisted with back-translation of responses into English.

We sent the questionnaire to 43 countries and received responses from 37 countries. The 6 countries that did not participate could not due to lack of funds, lack of permission from national authorities, or a need to attend to other priorities.

An in-country focal person from the Ministry of Health (MOH), MCHIP, or partner NGO led the national review and data collection in each country. These focal persons worked with local partners and stakeholders through a national consultative group to gather the necessary information and data and to complete the questionnaire. The consultative group was typically a maternal health working group convened by the government, with representation from relevant departments of the MOH, development partners, and implementing agencies.

Through an iterative series of participatory meetings, the partners reviewed the questions, provided responses, sought additional data for unanswered questions, and finally confirmed the responses on the completed instrument. We asked the groups to use nationally relevant documents, such as policies, the national essential medicines list (EML), service delivery guidelines (SDGs), and clinical standards, to respond objectively and with sufficient detail. Since the experts gathered in these consultative meetings are the people who would be at the forefront of policy implementation and practice in their countries, the information provided by them represents the most reliable and valid data available for the questions posed. Clarification was provided by the MCHIP/Washington team as required. Survey responses were sent to the MCHIP/Washington office where data were reviewed and cleaned. Data were entered into a Microsoft Access database to facilitate ease of data entry and analysis.

We also asked countries to provide copies of national guidelines and SDGs, and we analyzed national SDGs from 20 countries to determine the accuracy of survey results compared with the SDGs, as well as of national guidelines compared with global guidelines as the standard of reference. (See a list of documents reviewed in the supplementary material.) Using a standardized checklist adapted from WHO's *Managing Complications in Pregnancy and Childbirth: A Guide for Midwives and Doctors*,^9^ we focused on the following components in the SDG review: practice of AMTSL (including use of uterotonics); use of misoprostol for the prevention of PPH; diagnosis and management of PE/E, including the use of magnesium sulfate; and use of antihypertensives for severe hypertension in pregnancy.

Twelve SDGs were in English and were independently reviewed by the research team. The remaining 8 SDGs were in French and Spanish and were reviewed with an MCHIP country representative using a shorter checklist. Only questions asked of all countries about SDGs are included in the final sub-analysis. (See supplementary material for the checklists.)

Ethical clearance was not required because the survey reported on publically available information and respondents' responses were not recorded or individually reported.

## RESULTS

Results are organized according to policy and program elements deemed necessary by global recommendations for successful PPH and PE/E programming.

### Availability of Uterotonics (Oxytocin and Misoprostol)

**Figure 1.**
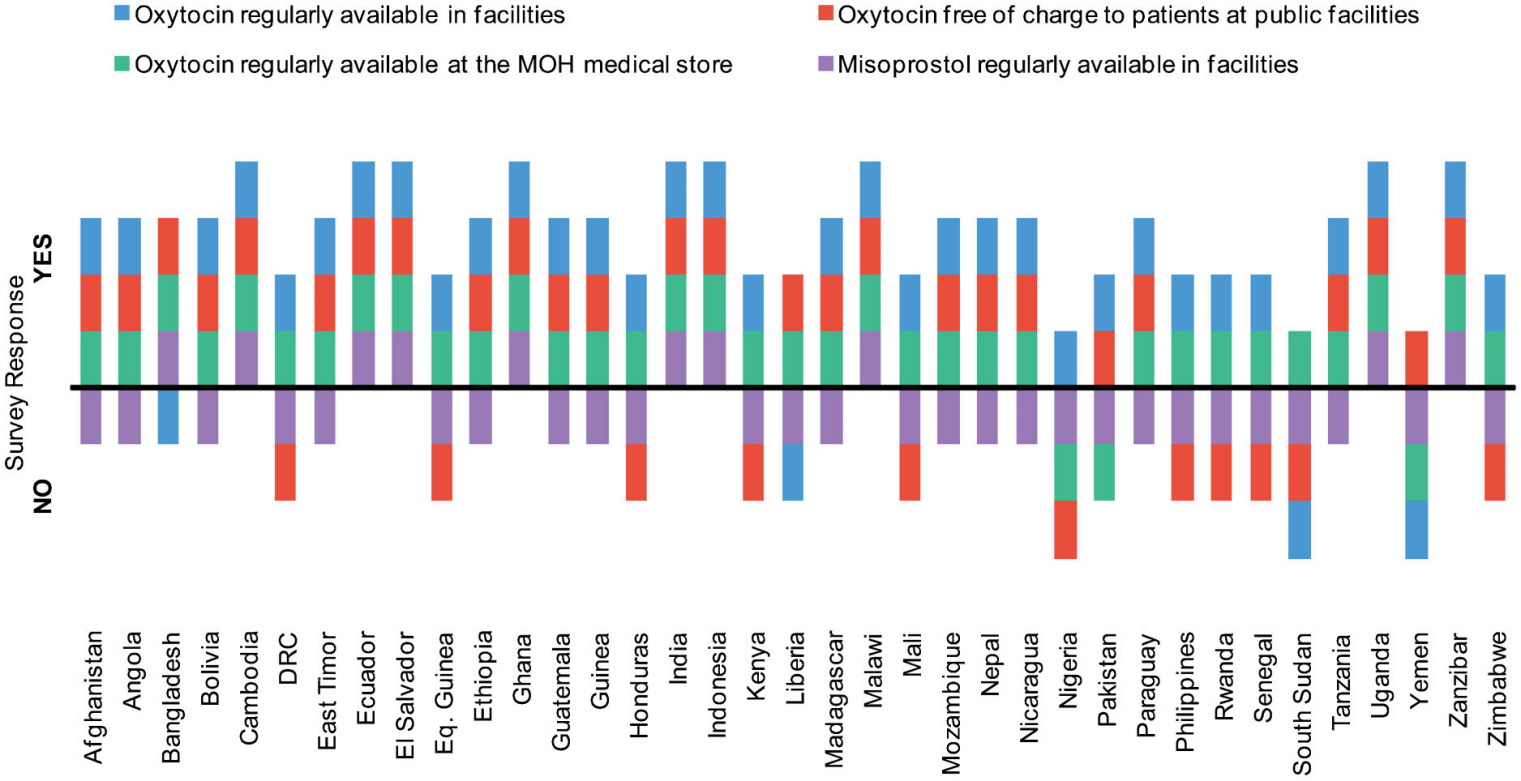
Availability of Uterotonics, 37 Surveyed Countries, 2012 Abbreviations: DRC, Democratic Republic of Congo; MOH, Ministry of Health.

Among the 37 countries surveyed, 33 countries (89%) reported regular availability (available “more than half the time”) of oxytocin *in facilities*, while only 10 countries (27%) reported regular availability of misoprostol (Figure 1). The 4 countries that reported that oxytocin was not regularly available were Bangladesh, Liberia, South Sudan, and Yemen. The vast majority of countries (34 of 37, or 92%) reported regular availability of oxytocin *in the national medical store or warehouse*. Countries that reported infrequent availability of misoprostol noted a lack of a national policy supporting misoprostol as a principle cause.

Oxytocin was regularly available in more countries than misoprostol.

About 70% of the countries (26 of 37) reported that oxytocin was provided for free to clients at public health facilities (Figure 1). In 9 countries, however, respondents reported that clients sometimes had to pay for oxytocin, even though national policy indicates that it should be provided at no cost. Illustrative follow-up responses explained why oxytocin is not always free to clients:

It is free of cost, whenever available. Most of the time it is not available and patients have to buy it or it is provided through charity/donation but not refrigerated.

If the Medical Supply at the Ministry distributes it, it will be free. But most of the time, it may not be there, as the amount distributed to health facilities is not sufficient. If it is not available, the family may buy it from the private pharmacy.

It was not specified whether this payment was for oxytocin inside the facility or for purchasing oxytocin outside the facility.

### Availability of Magnesium Sulfate

**Figure 2.**
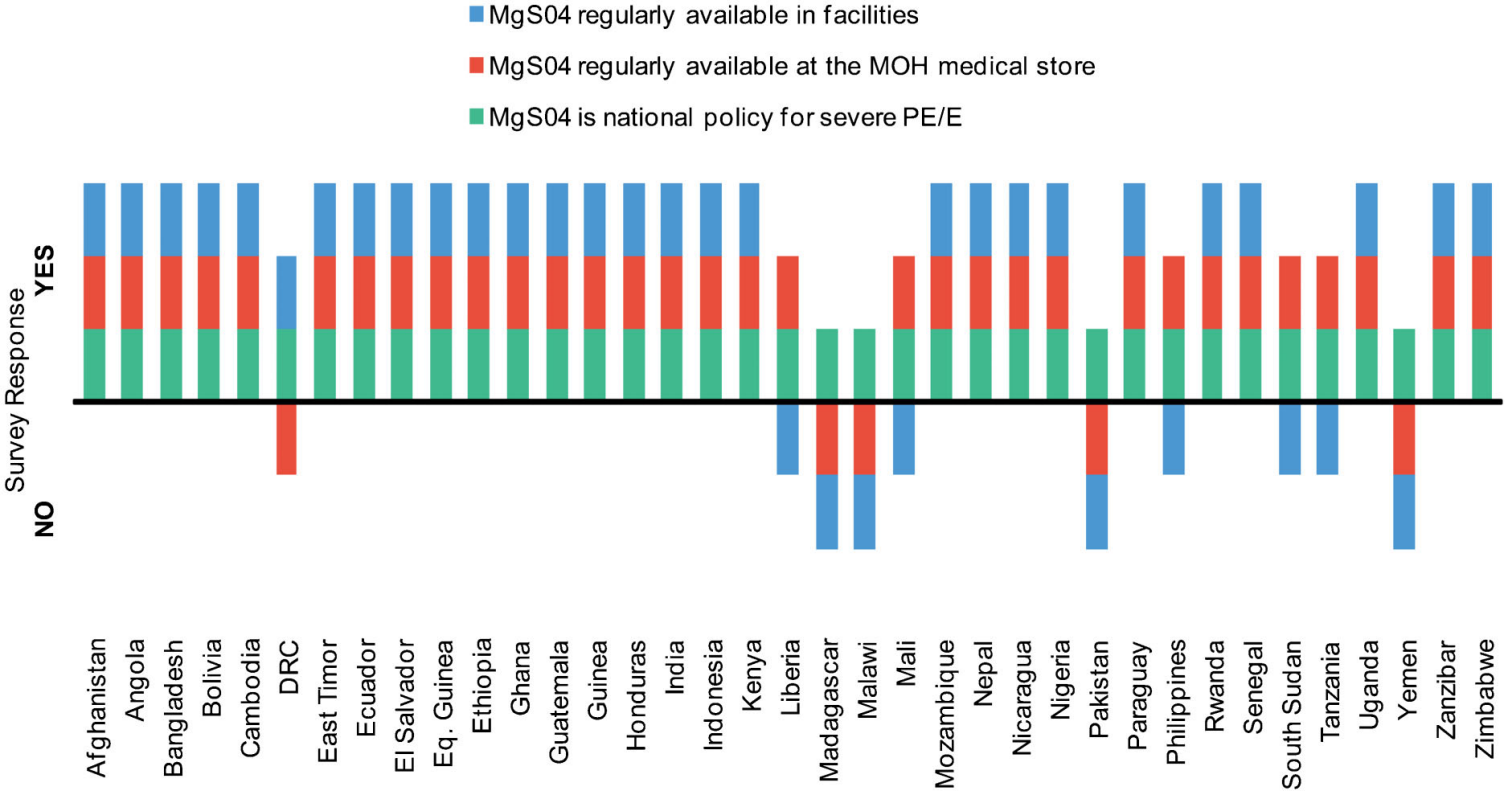
Availability of Magnesium Sulfate, 37 Surveyed Countries, 2012 Abbreviations: DRC, Democratic Republic of Congo; MOH, Ministry of Health; MgSO4, magnesium sulfate; PE/E, preeclampsia/eclampsia.

Most surveyed countries (28 of 37, or 76%) reported regular availability of magnesium sulfate in facilities (Figure 2). Of the 9 countries that reported that magnesium sulfate is still not regularly available at least half the time, 6 were in Africa and 3 in Asia. More countries (32 of 37, or 86%) reported that the medicine was regularly available in the MOH central medical store. Among surveyed countries, 46% reported that stock-outs of magnesium sulfate were rare, and an additional 46% reported that stock-outs occurred sometimes or were frequent.

### Medicines Approved at the National Level

**Figure 3.**
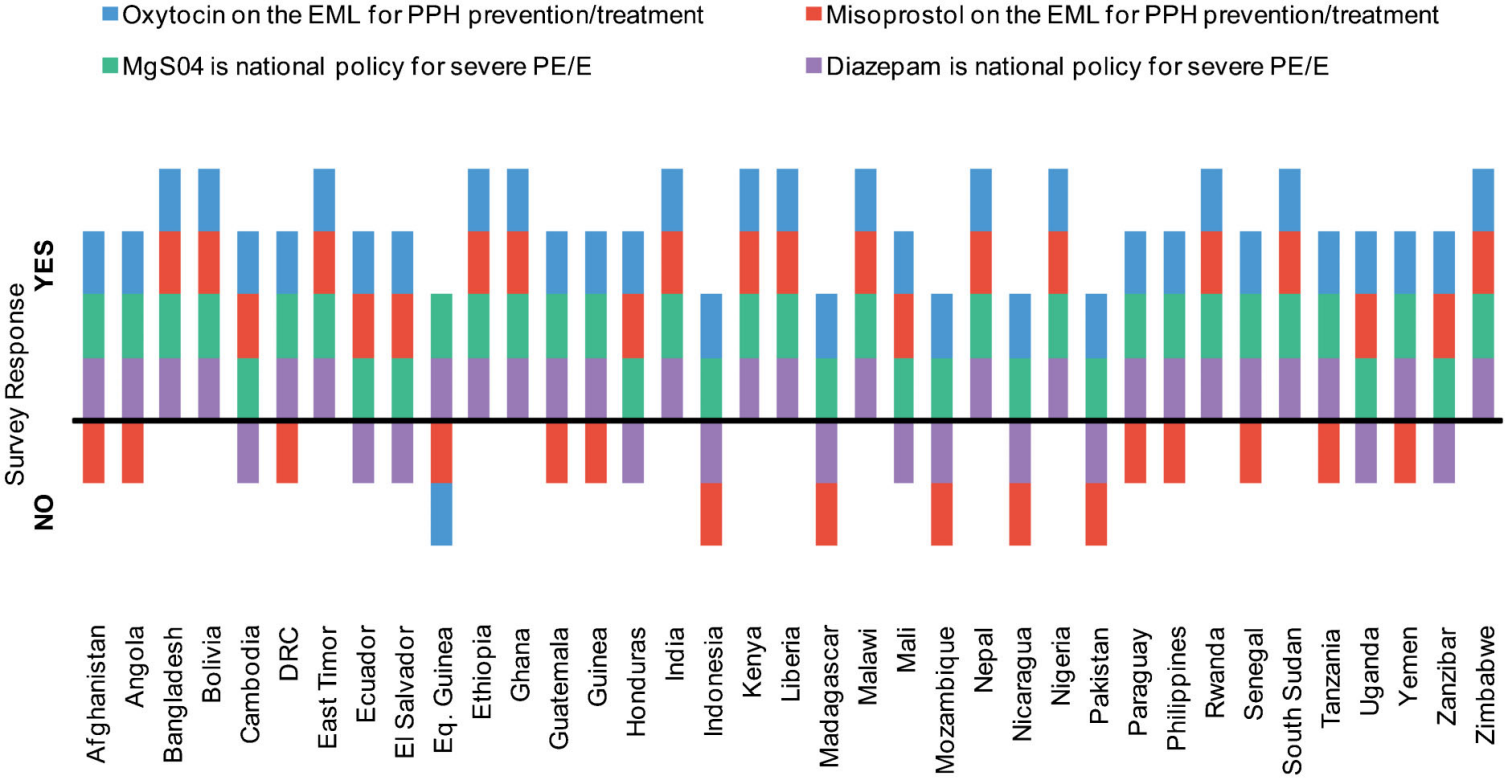
Medicines Approved at the National Level, 37 Surveyed Countries, 2012 Abbreviations: DRC, Democratic Republic of Congo; EML, essential medicines list; MgSO4, magnesium sulfate; PE/E, preeclampsia/eclampsia; PPH, postpartum hemorrhage.

All countries surveyed, except Equatorial Guinea, responded that oxytocin was on the EML for prevention/treatment of PPH (Figure 3). Only 21 of 37 countries (57%), however, reported that misoprostol was on the EML for prevention/treatment of PPH.

More countries included oxytocin on their essential medicines list than misoprostol.

All countries surveyed reported that magnesium sulfate is approved in national policy as a first-line anticonvulsant treatment for severe PE/E. A substantial number of countries (25 of 37), however, also included diazepam for the same indication. Of the 20 SDGs reviewed, 6 showed incomplete or inaccurate instructions for the use of magnesium in severe PE/E, compared with the WHO standard protocol for use of the drug.^9^

### Current Practice and Tracking of AMTSL

**Figure 4.**
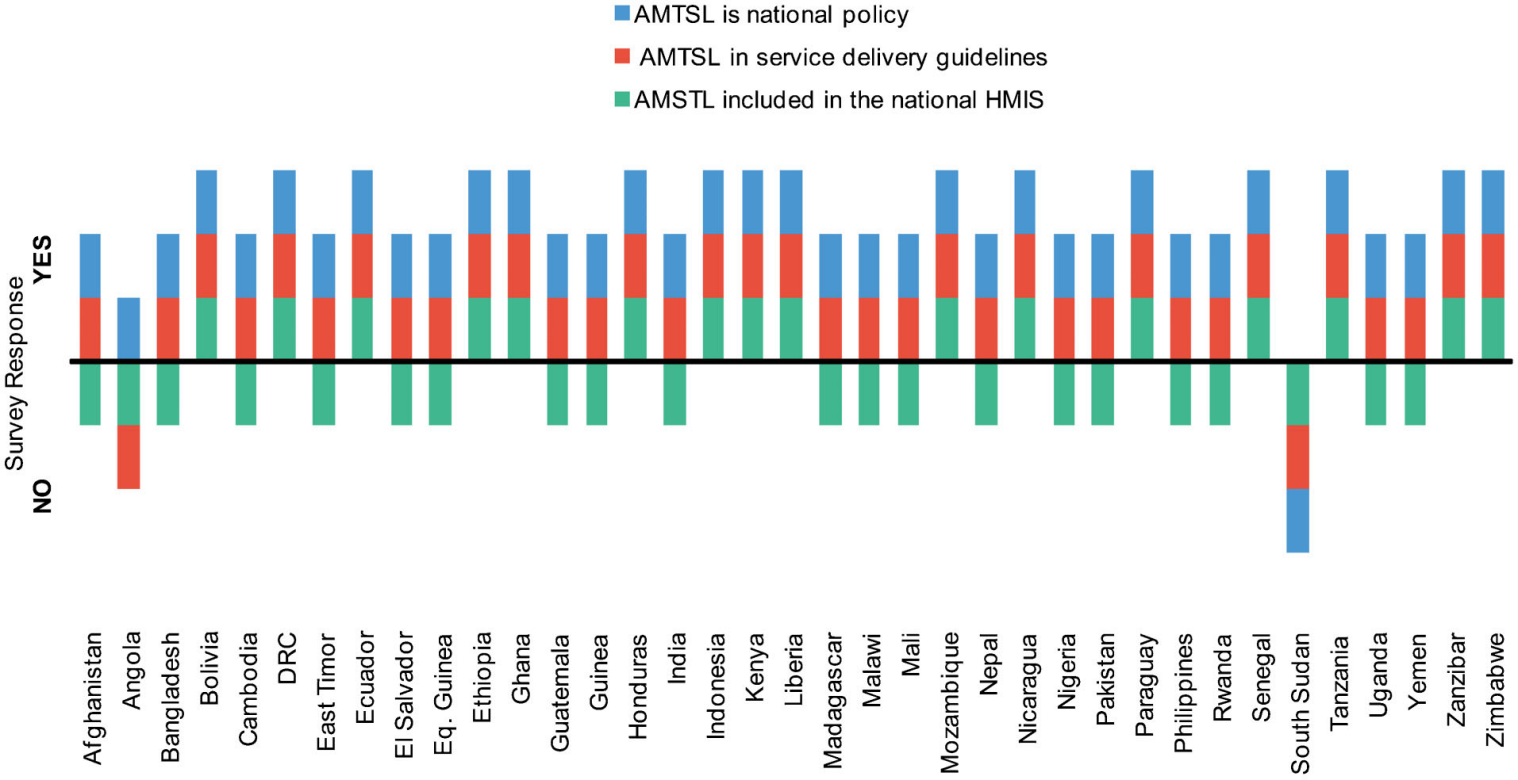
National Policy and Guidelines on AMTSL, 37 Surveyed Countries, 2012 Abbreviations: AMTSL, active management of the third stage of labor; DRC, Democratic Republic of Congo; HMIS, health management information system.

All but 1 surveyed country (South Sudan) reported that AMTSL was approved as national policy; 35 of 37 reported that it was included in the national SDGs (Figure 4). The focused review of national SDGs showed that all 20 SDGs included oxytocin as part of AMTSL; 18 of 20 indicated the correct dose of the medicine, but only 9 of the 20 contained accurate descriptions of the 3 essential components of AMTSL. A minority of surveyed countries (16 of 37) reported tracking AMTSL in their national health management information system (HMIS).

### Piloting Misoprostol for Home Birth

**Figure 5.**
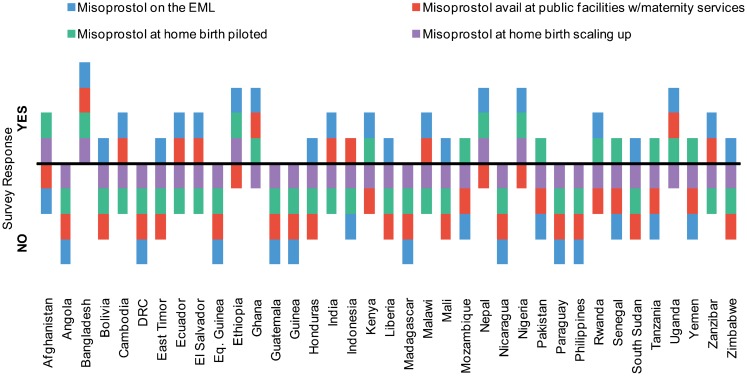
Availability and Use of Misoprostol, 37 Surveyed Countries, 2012 Abbreviations: DRC, Democratic Republic of Congo; EML, essential medicines list.

Sixteen of 37 countries (43%) reported that they were piloting or had piloted misoprostol for prevention of PPH at home birth; only 5 of 37, however, reported efforts to take this program to national scale (Figure 5). In follow-up qualitative responses, 7 countries reported that their governments do not support misoprostol for use at home births.

Some countries have piloted use of misoprostol to prevent PPH at home birth, but far fewer have taken the strategy to scale.

### Scope of Practice for Midwives/Skilled Birth Attendants

**Figure 6.**
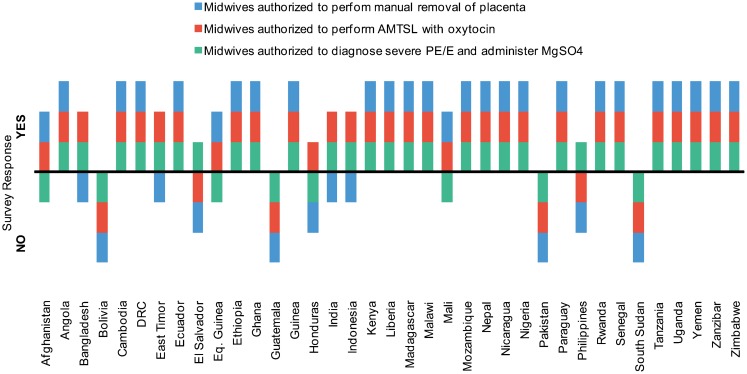
Midwifery Scope of Practice, 37 Surveyed Countries, 2012 Abbreviations: AMTSL, active management of the third stage of labor; DRC, Democratic Republic of Congo; MgSO4, magnesium sulfate; PE/E, preeclampsia/eclampsia.

Most countries (31 of 37, or 84%) reported that midwives/skilled birth attendants (SBAs) were authorized to perform AMTSL, including administration of oxytocin (Figure 6). Fewer (29 of 37, or 78%) reported that midwives/SBAs were authorized to diagnose severe PE/E and to administer magnesium sulfate to treat the condition. Fewer still (26 of 37, or 70%) reported that midwives/SBAs were authorized to perform manual removal of the placenta.

**Figure:**
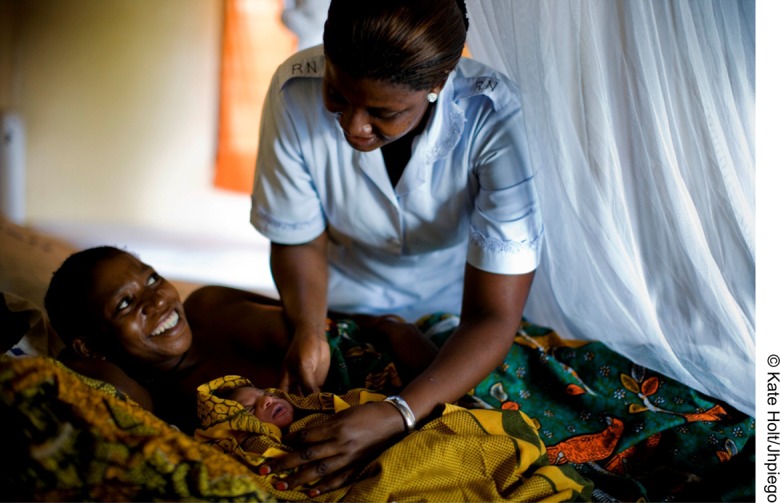
A midwife in Tanzania checks in on a mother and her newborn baby.

## DISCUSSION

Overall, results of this key informant survey suggest that most countries are appropriately prioritizing policies and practices that are essential for strong PPH and PE/E programs. Issues remain, however, that need to be addressed.

### Availability of Essential Medicines

Inconsistent availability of essential medicines limits implementation of national priorities and can lead to inadequate clinical management. Despite general approval of oxytocin as an essential medicine for preventing PPH, challenges with free distribution, proper storage, and maintaining a regular supply of the medicine persist. This suggests a need for better supply chain management of maternal health medicines and supplies, as well as greater coordination between clinical/service provision, the central medical stores, and supply chain management and logistics departments of health ministries. This type of key informant survey cannot address the additional issue of oxytocin potency. It must be recognized, however, that heat instability and oxytocin deterioration can be an additional and critical dimension in any consideration of uterotonic availability.

Better supply chain management of maternal health medicines and supplies is needed.

Magnesium sulfate has global approval as an essential medicine for managing PE/E and its consequences. This was similarly reflected in our multi-country survey. As with oxytocin, however, free distribution and maintenance of a regular supply of the medicines are ongoing challenges that limit success despite universal endorsement. Qualitative responses to the survey reveal that for PE/E in particular, lack of regular magnesium sulfate availability is one of the most critical barriers to scaling up the intervention. Strengthening the supply chain for magnesium sulfate, ensuring that all SBAs are permitted and competent to use it, and identifying and addressing additional barriers are necessary components of national programs.

Likewise, although misoprostol is known to be effective in preventing PPH, most of the surveyed countries reported limited availability, both in facilities and at the national store. Although the use of misoprostol to prevent PPH at home birth has been piloted in many countries,^10^ in this survey only 5 of the 16 countries that have piloted the strategy have moved toward scale up—Afghanistan, Bangladesh, Ethiopia, Nepal, and Nigeria. This disconnect between pilot and scale up is concerning and reflects lingering skepticism about the place of this important intervention in maternal health programming. This could be due in part to conflicting global guidance, which has, until recently, limited countries' willingness to proceed. In 2012, however, WHO revised its PPH guidelines to state that “when skilled birth attendants are not present and oxytocin is unavailable, community health care and lay health workers should administer misoprostol (600 *μ*g PO) for PPH prevention.” These new recommendations may result in changes to some national program strategies in the coming years. Additionally, the inclusion of misoprostol as one of the 13 essential commodities of the UN Commission on Life-Saving Commodities for Women and Children (UNCoLSC) has given global prominence to this medicine. Our survey findings suggest an opportunity for global action and advocacy, especially given the growing support for programs to prevent PPH using misoprostol and the research that continues to emerge.

Barriers to access and availability of key maternal health medicines where they are needed are now being addressed under the high-level UNCoLSC, which is leading advocacy and policy efforts to ensure sufficient supply, quality, and use at the country level.^11^ Paying for medicines is a bottleneck to improving coverage of high-impact interventions, despite the fact that the 3 lifesaving commodities addressed in the survey are considered to be inexpensive. As countries accelerate progress toward Millennium Development Goal 5 (improve maternal health), greater emphasis must be given to equity and the need to focus efforts on reaching the poorest and most vulnerable groups.^12^

### Up-to-Date Service Delivery Guidelines

Acceptance of the use of uterotonics (and AMTSL) as a routine part of care during childbirth is nearly universal. New evidence has highlighted the central importance of the administration of a uterotonic in the prevention of PPH.^13^ However, technical inconsistencies in national SDGs must be addressed. National guidelines were sometimes incomplete or out-of-date, a fact that sometimes conflicted with respondents' answers to the survey. This suggests that stakeholders may perceive their country guidelines to be more accurate than they in fact are. Such discrepancies may be expected as national SDGs try to keep pace with the advancing and evolving global evidence. Efforts must be made, however, to disseminate new information and to support countries as they revise existing guidelines.

National service delivery guidelines must be updated continually to keep pace with the evolving global evidence.

### Reporting Systems

Lack of national reporting in the HMIS on use of uterotonics (and AMTSL), as well as other key indicators related to maternal health, currently limits and will continue to constrain progress. Gathering sufficient data on implementation of critical interventions is important to ensure that these interventions are prioritized and that progress is measured.

### Scope of Practice for Midwives

A “scope of practice” defines the responsibilities and activities that a licensed practitioner is permitted to perform in health care, per national policy. Although there has been some progress in expanding midwifery scopes of practice, not all countries include all 7 basic emergency obstetric and newborn care (BEmONC) signal functions within that scope,* despite the fact that they are included in the *Essential Competencies for Basic Midwifery Practice*^6^ of the International Confederation of Midwives (ICM) and listed as essential interventions by WHO and others.^14^^,^^15^ Our survey results confirm that the role of the midwife varies by country and that midwives have a larger scope of practice in Asia and Africa than in Latin America, where in Bolivia, Guatemala, and Honduras, midwives are not allowed to perform AMTSL.

Midwives should be permitted to perform all 7 basic emergency obstetric and newborn care functions.

If the need for an emergency maternal health intervention exceeds the availability or capacity of service providers to provide it, women's lives are at risk. When there are complications during childbirth, a midwife or SBA needs to be both competent and authorized to perform all 7 BEmONC skills. There is strong and increasing support for a scope of practice for midwives that will allow them to provide the services needed to reduce the main causes of maternal mortality, including endorsements by WHO, the United Nations Population Fund (UNFPA), ICM, and the United Nations Children's Fund (UNICEF).^15^ In the qualitative responses in this survey, task shifting and supportive policies were also reinforced as essential for program scale up.

### Limitations

This survey had several limitations, despite efforts to ensure that the survey was as objective as possible. In some cases, country respondents may not have had complete information or data, or full access to such information or data, to allow for thorough, objective quantitative responses, for example, records on commodity stocks. Therefore, stock-outs may have been underestimated in this study. Additionally, there was little information on the representatives in the stakeholder group, specifically MOH involvement, although this was actively promoted. Qualitative responses provided valuable information and helped to triangulate the quantitative responses, but they are based on opinion and may or may not represent the majority opinion of health professionals in a particular country. There were often gaps in answers about approved medicines for PE/E. When possible, the research team worked with countries to fill these gaps, and this may have affected objectivity. Many countries use different terms for the same activity or process and during attempts to clarify these differences, certain nuances may have been lost.

## CONCLUSION

This survey offers opportunities to review national programs for addressing PPH and PE/E. It provides a multi-country snapshot of policy, practice, supplies, and activities, and guides national and global program managers and policy makers in setting priorities.

Recommended actions, based on the available data, include:

- Increased support for using misoprostol to prevent PPH at home births to allow greater coverage of uterotonic use in the third stage of labor than allowed by facility-based oxytocin alone. This is especially important in areas with high home birth rates and low skilled birth attendance.
- Additional effort to ensure availability and use of magnesium sulfate, as part of appropriate and comprehensive management of women with PE/E
- Clear definition of and uniform application of the midwifery scope of practice, consistent with the ICM's recognized essential competencies and the international definition of a midwife^6^^,^^16^
- Updating national clinical guidelines and essential medicines lists to be consistent with WHO recommendations for PPH and PE/E
- Revision of health monitoring and reporting systems to include and track key interventions in maternal health

As countries scale up prevention and treatment programs for PPH and PE/E, national programs will need to ensure both adequate coverage and sustainability, and tracking the progress of national programs while supporting greater efforts to reduce maternal morbidity and mortality will be essential.

## Footnotes

* BEmONC includes 7 signal functions: administer parenteral antibiotics; administer uterotonic drugs; administer parenteral anticonvulsants; perform manual removal of placenta; perform removal of retained products (for example, manual vacuum aspiration); perform assisted vaginal delivery (for example, vacuum extraction); perform neonatal resuscitation.

## Notes

**Peer Reviewed**

**First Published Online:** 2014 Jul 3
